# Burden of disease due to cancer in Spain

**DOI:** 10.1186/1471-2458-9-42

**Published:** 2009-01-30

**Authors:** Nerea Fernández de Larrea-Baz, Elena Álvarez-Martín, Consuelo Morant-Ginestar, Ricard Gènova-Maleras, Ángel Gil, Beatriz Pérez-Gómez, Gonzalo López-Abente

**Affiliations:** 1 Health Technology Assessment Unit, Laín Entralgo Agency, Madrid Regional Health Council, Calle Gran Vía, 27, 28013 Madrid, Spain; 2 Preventive Medicine and Public Health Teaching and Research Unit, Faculty of Health Sciences, Rey Juan Carlos University, Madrid, Spain; 3 Department of Mental Health, Madrid Regional Health Council, Madrid, Spain; 4 Directorate General Public Health, Madrid Regional Health Council, Madrid, Spain; 5 Environmental and Cancer Epidemiology Unit, National Centre for Epidemiology, Carlos III Institute of Health, Madrid, Spain; 6 CIBER en Epidemiología y Salud Pública (CIBERESP), Spain

## Abstract

**Background:**

Burden of disease is a joint measure of mortality and morbidity which makes it easier to compare health problems in which these two components enjoy different degrees of relative importance. The objective of this study is ascertaining the burden of disease due to cancer in Spain via the calculation of disability-adjusted life years (DALYs).

**Methods:**

DALYs are the sum of years of life lost due to premature mortality and years lost due to disability. World Health Organization methodology and the following sources of data were used: the Mortality Register and Princeton Model Life Table for Years of life lost due to premature mortality and population, incidence estimates (Spanish tumour registries and fitting of generalized linear mixed models), duration (from data of survival in Spain from the EUROCARE-3 study and fitting of Weibull distribution function) and disability (weights published in the literature) for Years lost due to disability.

**Results:**

There were 828,997 DALYs due to cancer (20.5 DALYs/1,000 population), 61% in men. Of the total, 51% corresponded to lung, colorectal, breast, stomach and prostate cancers. Mortality (84% of DALYs) predominated over disability. Subjects aged under 20 years accounted for 1.6% and those aged over 70 years accounted for 30.1% of DALYs.

**Conclusion:**

Lung, colorectal and breast cancers are responsible for the highest number of DALYs in Spain. Even if the burden of disease due to cancer is predominantly caused by mortality, some cancers have a significant weight of disability. Information on 2000 burden of disease due to cancer can be useful to assess how it has evolved over time and the impact of medical advances on it in terms of mortality and disability.

## Background

Burden of disease (BoD) is a measure of population health which takes into account mortality and morbidity due to different diseases and injuries [1]. Disability-adjusted life years (DALYs) are used as a measure of BoD. This summary indicator represents the number of years of healthy life lost due to a disease or risk factor. It was first employed in the Global Burden of Disease (GBD) study undertaken by the World Health Organization (WHO) and Harvard University [2] in 1996; since then, DALYs have been also used to study BoD at national level [3-8].

The results of BoD studies are potentially an aid to public health decision making, to detect areas of improvement in information systems and to conduct cost-effectiveness studies. Furthermore, DALYs due to a disease can be applied to the calculation of DALYs due to a risk factor. For instance, DALYs due to tobacco related cancers are needed to calculate DALYs due to this exposure, and consequently to estimate the potential health gain expected from interventions that reduce tobacco consumption.

Cancer is one of the leading causes of morbidity and mortality in developed countries. In Spain, it was the second leading cause of death [9] in 2000 (and the leading cause in men). In 2003, there were three deaths per thousand men and 1.7 deaths per thousand women due to malignant tumours.

Cancer comprises a group of malignancies that, while having certain characteristics in common, have very different causes and show widely differing response to treatments. DALYs might be a useful tool for comparing the BoD of different tumour sites, which have a different relative weight of disability. Indeed they may be useful to study the evolution of the two components, mortality and disability, over time.

Cancer epidemiology has been extensively studied, mainly in terms of mortality. The increase in survival in many malignant neoplasms means that, in addition to mortality, it is increasingly necessary to take into account the non-mortal consequences of disease (disability due to the disease itself or to treatment, and the worsening of the patient's quality of life). Accordingly, the aim of this study was to estimate and analyse the burden of disease due to malignant neoplasms in Spain in 2000. In order to do this, disability-adjusted life years were used.

## Methods

DALYs were calculated by applying the methodological principles employed in the GBD study [10]. DALYs are the result of adding years of life lost due to premature mortality (YLL) to years lost due to disability (YLD). YLL due to cancer among all persons that die of cancer are the sum of years that they would have lived if they had completed the life expectancy attributed to their age at the time of their death. YLD express the consequences of living with a less than perfect health condition, and they are estimated based on the length of time with that condition and any accompanying disability. The variables needed to estimate DALYs were: mortality, incidence, duration, disability and age at diagnosis. To compute DALYs, the social values used in the GBD study [2], namely, discount rate (3%) and age weighting (K = 1), were applied. The computer programmes used were Gesmor [11] and DisMod [12].

Different data sources were used for each of the estimated parameters:

1) Population: National Institute of Statistics (Instituto Nacional de Estadística – INE). 2) Mortality: National Institute of Statistics data file, coded as per the International Classification of Diseases (ICD-10); the codes selected for each cancer are listed in Table 1. Deaths attributed to ICD-10 Group R ("ill-defined") were reassigned in accordance with GBD study criteria [2]. Likewise, deaths attributed to neoplasms of unspecified site (ICD-10 code C80) and metastasis (ICD-10 codes C77, C78 and C79) were redistributed among all malignant tumours. 3) Life expectancy: modified level 26 of the Princeton Model Life Table [13] was taken as the limit for calculating potential years of life attributed to each age. 4) Incidence: Spanish population-based cancer registries. 5) Disease duration: Survival data in Spain from the European Cancer Registries Study on Cancer Patients' Survival and Care (EUROCARE-3) [14] and the US Surveillance, Epidemiology and End Results (SEER) [15]. And 6) Disability weights: Disability Weights for Diseases in The Netherlands [16] and Victorian Burden of Disease Study [17].

**Table 1:** Epidemiological data used to calculate Disability-Adjusted Life Years

**CANCER SITE **(ICD-10 code)	**SEX**	**MORTALITY **(number of deaths) *	**INCIDENCE **(number of new cases) †	**DURATION **(years) ‡	**DISABILITY** (range 0–1) §
Bladder (C67)	Males	3438	12720	4.2	0.24
	
	Females	804	1750	3.7	0.31

Brain (C71)	Males	1331	1954	1.5	0.64
	
	Females	1046	287	1.4	0.66

Breast (C50)	Males	59	-	-	-
	
	Females	6206	15979	4.3	0.38

Colon and rectum	Males	6958	14204	3.4	0.37
	
(C18-C21)	Females	5740	11461	3.5	0.36

Gall bladder (C23-C24)	Males	517	696	1.6	0.46
	
	Females	1000	1542	1.4	0.49

Hodgkin's disease (C81)	Males	170	890	4.9	0.24
	
	Females	135	528	4.9	0.25

Kidney (C64-C66, C68)	Males	1184	1441	3.7	0.30
	
	Females	636	1189	3.7	0.30

Leukaemias (C91-C95)	Males	1750	2436	3.6	0.37
	
	Females	1386	1852	3.6	0.36

Liver (C22)	Males	2997	3081	1.1	0.38
	
	Females	1621	1309	0.9	0.45

Lung (C33-C34)	Males	16629	16690	1.4	0.68
	
	Females	2056	2131	1.6	0.67

Melanoma (C43)	Males	426	1283	3.9	0.22
	
	Females	339	1785	4.6	0.19

Myeloma (C90)	Males	827	795	3.4	0.27
	
	Females	863	769	5.5	0.24

Non-Hodgkin's lymphoma	Males	1341	3253	4.2	0.31
	
(C82-C85, C96)	Females	1211	2209	4.2	0.31

Oesophagus (C15)	Males	1675	1512	1.4	0.76
	
	Females	260	257	1.6	0.78

Ovary (C56-C57)	Females	1759	2997	3.1	0.34

Pancreas (C25)	Males	2231	1919	0.7	0.64
	
	Females	2117	1675	0.7	0.63

Prostate (C61)	Males	5894	13212	3.1	0.43

Stomach (C16)	Males	4038	2896	2.3	0.62
	
	Females	2592	3454	2.0	0.60

Thyroid (C73)	Males	116	456	4.6	0.22
	
	Females	209	1278	4.7	0.22

Uterus and cervix (C53-C55)	Females	1975	7164	4.2	0.26

Other malignant neoplasms	Males	7137			
	
	Females	3179			

ICD-10: International Classification of Diseases, 10th Revision.

* Source: National Institute of Statistics Spain (INE). National cause-of-death register, year 2000.

† Source: estimated from incidence data from regional population-based cancer registries.

‡ Source: estimated from relative survival data from the EUROCARE-3 study. Data are averages of duration in patients who die of cancer and in patients cured, weighted by the cure rate.

§Source: ref. 16, 17. Data are averages of the disability weight of each disease stage, weighted by its duration and then by the cure rate.

Spanish 2000 mid-year population: 19,778,785 males and 20,597,509 females (Source: National Institute of Statistics, INE).

**Figure 1:**
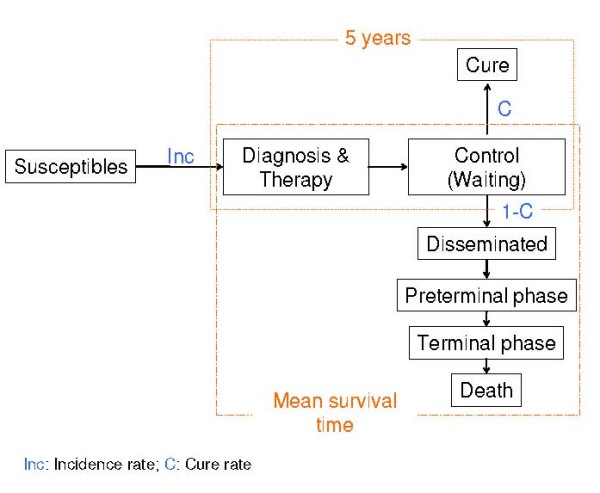
**Diagram of disease model for estimating cancer-related Years Lost due to Disability**. The model differentiates two groups of patients: those who do not die from cancer (C) and those who die from cancer (1-C). Mean survival time for the latter is estimated by fitting Weibull distribution to survival data.

The disease model relied on the WHO-designed model [18], using only the part corresponding to cancers that received treatment. It was a simplification of the disease history, describing the different stages. Duration and disability weights would be assigned for each stage. In some tumours, the model subdivided groups by stage or size of tumour at diagnosis and/or histological type (see additional file 1: Distribution of cancer stages at diagnosis). The model (Figure 1) differentiated two possible cancer evolutions: patients who would be cured of cancer and those who would die of cancer. The cure rate was estimated from the cumulative relative survival until a predefined cure threshold (the time point from which a person could be deemed to be cured). This cure threshold was established for each cancer site as the number of years in which interval-specific relative survival was 100% since diagnosis was made [19,20].

Incidence from 1997 to 2000 was estimated [9] on the basis of cancer incidence data for the period 1983–1997. Generalized linear mixed models were fitted, based on incidence-mortality ratio. The dependent variable was the incidence/mortality ratio and the independent variables were age, period and province of residence. Province was included as a random-effect term to take provincial heterogeneity into account. Model parameters were obtained by Bayesian methods, using WinBugs [21].

The distribution percentages of incident cases in the different subgroups were obtained from published studies conducted in Spain [22,23], except in the case of melanoma and leukaemias and lymphomas (which were drawn from the Australian GBD study, after confirming its similarity with data from Spain's 1999 Minimum Basic Hospital Data Set [24]).

Disease duration was estimated separately for the group of patients who were cured and for the group of patients who died from cancer. The duration of disease was set at 5 years, following the WHO disease model, for the first group and it was estimated by applying the WHO methodology [18] for the second group. On the assumption that time of survival followed a Weibull distribution, this time was estimated by adjusting survival data (at 1, 3 and 10 years from diagnosis) to this distribution function [18,25]. As survival data at 10 years were incomplete in Spain, these were estimated by applying the ratio between survival at 5 and 10 years in the USA [15], to survival at 5 years in Spain. Mean disease duration by age group and sex was calculated as the mean of disease duration in the two groups, weighted by the cure rate. The duration of each disease stage was based on the Victorian BoD study [17]: this establishes the duration of all stages but one, which is then calculated as the difference between the estimated total duration and the sum of the durations of all the other stages.

A disability weight of 0 (perfect health) to 1 (death) was applied to each of the stages through which each type of cancer goes through. For cancers in which the disease model is divided in subgroups with different disability weights, global disability was calculated as the weighted mean according to: the duration of each stage in each subgroup, the frequency of each subgroup in the population and the cure rate.

The midpoint of each age group was considered as the age of diagnosis. In the open age group (over 85 years of age), midpoint is defined by the life expectancy at 85 years, so these figures were considered (91.22 years among women and 90.24 among men).

Cancers that were not analysed individually were included in the "Other malignant tumours" group. In this case, YLD were estimated for each age and sex groups applying the combined YLL/YLD ratio for all the other cancers to the YLL of the "Other malignant tumours" group according to the WHO methodology [26].

## Results

The intermediate results (mortality, incidence, duration and disability) needed to estimate DALYs are set out in Table 1. Cure rate estimates can be seen on the additional file 2: Cure thresholds and cure rates.

The number of DALYs lost due to cancer was 828,997 in Spain in 2000 (61% in men). There were a total of 698,271 YLL (63% in men) and 130,726 YLD (54% in men).

By tumour site, neoplasm of lung was responsible for the greatest number of DALYs (20% of the total), with colon and rectum's in second place (12%) and breast neoplasm in third place (9%). These three tumour sites, along with neoplasms of stomach, prostate, bladder, liver, pancreas and leukaemias account for more than two thirds of the total burden of disease due to cancer. Table 2 shows BoD of different cancer sites, in males and females.

**Table 2:** Disability-Adjusted Life Years by cancer site and sex

**Females**			**Males**		
**CANCER SITE**	**DALY**	**DALY/100,000 population**	**CANCER SITE**	**DALY**	**DALY/100,000 population**

Breast	76944	374	Lung	145499	736

Colon & rectum	43671	212	Colon & rectum	56162	284

Uterus	23217	113	Prostate	33459	169

Lung	20111	98	Stomach	31545	159

Stomach	18427	89	Bladder	28176	142

Ovary	18234	89	Liver	22176	112

Pancreas	13688	66	Leukaemias	18646	94

Leukaemias	13685	66	Pancreas	17745	90

NHL	11342	55	Brain	16908	85

Brain	10912	53	Oesophagus	16587	84

Liver	9664	47	NHL	15458	78

Gall bladder	6083	30	Kidney	10016	51

Myeloma	5667	28	Myeloma	5928	30

Kidney	5494	27	Melanoma	5476	28

Bladder	4656	23	HD	3584	18

Melanoma	4510	22	Gall bladder	3352	17

HD	2357	11	Thyroid	1267	6

Thyroid	2300	11	Breast	438	2

Oesophagus	1850	9	Other	76760	388

Other	27003	131			

TOTAL	319815	1553	TOTAL	509182	2574

Total Spanish 2000 mid-year population: 19,778,785 males and 20,597,509 females (Source: National Institute of Statistics).

DALY: Disability-Adjusted Life Years; NHL: Non-Hodgkin's lymphoma; HD: Hodgkin's disease.

**Figure 2:**
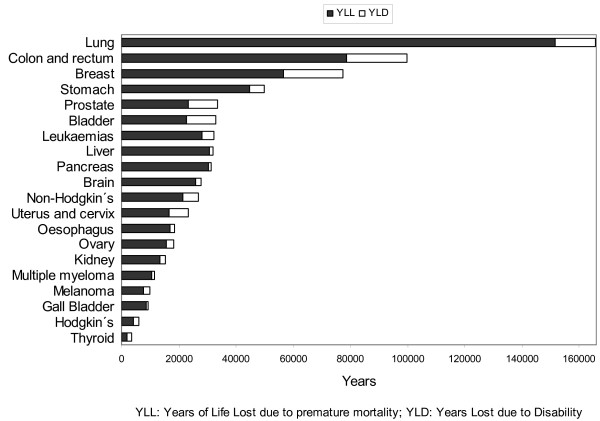
**Cancer burden of disease in Spain in 2000**. Disability-Adjusted Life Years are the sum of YLL and YLD. The figure shows the distribution of premature mortality (YLL) and disability (YLD) by cancer site.

Analysing the two components of DALYs, lung cancer accounted for the highest number of YLL (22% of all YLL due to cancer) and colorectal cancer accounted for most years lost due to disability (16% of all YLD due to cancer). Figure 2 depicts the respective weights of the two BoD components (mortality-YLL and morbidity-YLD) for each neoplasm. While mortality outweighed disability in the overall cancer burden, the two components were most equally balanced in malignant neoplasm of thyroid gland (YLL/YLD ratio: 1.2). Cancer of the bladder, prostate and uterus and Hodgkin's disease (HD) registered a YLL/YLD ratio of 2. Pancreatic and liver cancers were lying at the opposite extreme (YLL/YLD: 25). Overall, YLD accounted for 16% of DALYs (14% in men and 19% in women).

**Figure 3:**
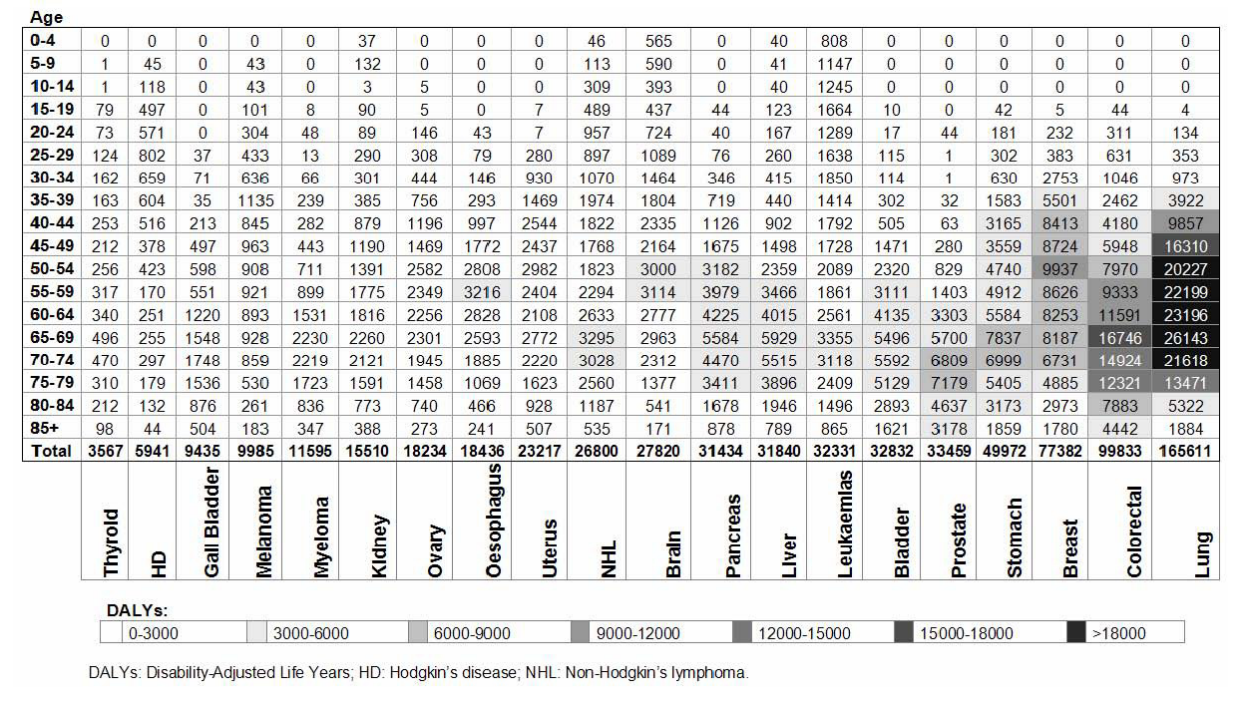
**Distribution of Disability-Adjusted Life Years in 5-year age groups. Spain 2000**.

In terms of the age distribution, patients aged 65–69 years registered the highest number of DALYs. Figure 3 represents the number of DALYs lost due to each cancer at each age. The general pattern showed a concentration of DALYs from the forties onwards, rising progressively to reach a peak in the five-year period from 65 to 69 years, except in cancers of bladder and gall bladder (70–74 years) and prostate (75–79 years). Some cancers, such as gynaecological tumours and melanoma, were characterized by an earlier rise in DALYs, with the peak in the 50–54-age-group or earlier. Hodgkin's disease (HD) had a characteristic pattern, with a peak in young adults (aged 25–29 years). Overall, patients under the age of 40 lost 9% of all DALYs due to cancer. Leukaemias registered the highest percentage of DALYs from ages 0 to 19 years (15%).

## Discussion

In 2000, the burden of disease due to cancer was 828,997 DALYs in Spain (21 years of disability-adjusted life lost per 1,000 population). This figure is consistent with that of 21 DALYs/1,000 population calculated by the WHO for the Euro-A Region in 2000 [27]. Cancers account for 16% of the BoD in Spain [28], the same as in the Euro-A Region [27] and they are the second leading cause of DALYs, after neuropsychiatric disorders (28% of the total BoD).

In Spain, the greatest BoD due to cancer is attributable to lung, colorectal, breast, stomach and prostate cancers. The high BoD of lung and colorectal cancers is due both to their incidence and mortality. Even though breast cancer causes fewer deaths, it has a high incidence (almost as high as the incidence of lung cancer in men) and it is the leading cause of cancer-related DALYs among women. Malignant neoplasm of stomach continues to be an important cause of BoD in the Spanish population, despite the fall in its incidence and mortality in the second half of the 20th century [9]. The elevated BoD of prostate cancer is mainly due to its high incidence and long duration, factors which make it one of the weightiest cancers in terms of disability.

The ranking of cancers according to DALYs is similar to that according to mortality, as it was expected given the lethality of these diseases. However, there are some differences between these rankings. The cancer sites that gain more positions in the ranking according to DALYs are bladder cancer and leukaemias (6^th ^and 7^th ^respectively, increasing from 8^th ^and 9^th ^according to mortality). Conversely, those that lost more positions are liver and pancreas cancer (8^th ^and 9^th ^respectively according to DALYs and 6^th ^and 7^th ^according to mortality).

In all cancer sites, the mortality component gives more weight to the BoD than the disability component. However, in some cancer sites such as thyroid, breast, bladder, uterus, prostate and in Hodgkin's disease, more than 25% of its BoD is due to YLD. This highlights the need to consider the effects of cancer interventions on disability as well as on mortality when assessing them.

Young people have a greater relative weight according to cancer BoD estimates than according to cancer mortality data: people under the age of 40 lost 9% of all cancer DALYs, compared to 2% of all cancer deaths.

The fall in DALYs after ages 65–69 years is due to the lower number of persons at these ages and to the fewer YLL per death. In malignant neoplasms of gall bladder and prostate, older age groups register maximum BoD. Therefore, a special increase in this particular BoD is to be expected in the future as a consequence of the ageing of the population. In malignant neoplasms of brain, gynaecological tumours, melanoma, leukaemias and HD, the age distribution of DALYs reflects an earlier incidence than in other tumours and, in some cases, a greater aggressiveness of the disease at younger ages. In the case of breast cancer, it is worthy a note that 34% of its BoD is experienced by women under 50 years and 21% by women older than 69. Breast cancer screening programmes are targeted to women between 50 and 69 years of age in most of the Spanish regions. In colorectal cancer, 10% of DALYs are lost by patients between 40 and 50 years, 27% between 70 and 80 years and 12% by older than 80 years. It is also remarkable that 45% of the BoD attributable to melanoma is experienced by persons under the age of 50 years (11% between 35 and 39 years). In cancers for which population based screening programmes are feasible, information about the age distribution of their BoD could be useful for the identification of the optimal target population.

There are differences in the epidemiology of cancer between men and women. As it has been reported elsewhere [3,27], men register a higher number of DALYs due to cancer than women. Neoplasms of oesophagus, lung and bladder predominantly affect men, who register over 80% of all DALYs for these three sites. This can be explained by the great weight of smoking as a risk factor for these cancers and by the higher prevalence of smoking among men in the past [29,30]. The acquisition of smoking habit by women began later [31], but has reached even higher levels; thus, it is likely that in the next years BoD due to smoking-related cancers will increase among women. Malignant tumours of liver, kidney, stomach and brain also cause a greater BoD in men. In contrast, neoplasms of breast, thyroid and gall bladder are responsible for a greater BoD in women.

The disability component in cancer BoD is greater among women than among men. This is probably due to the higher frequency of lung and oesophageal cancer in men, both of which are highly lethal and short in duration, and of breast, thyroid and uterus cancer in women, all of which are less lethal and of longer duration.

In the Australian [3] and New Zealand BoD studies [4], the three cancers that account for the heaviest BoD are the same as in Spain. Stomach cancer, however, ranks tenth and seventh respectively, and neither bladder nor liver cancer ranks among the top ten, as is the case in Spain. The relatively high incidence of bladder tumours in Spain has been previously described [9]. The relative weight of melanoma in New Zealand's BoD is greater than it is in Spain. In the Euro-A Region [27], BoD due to cancer is similar to that of Spain, with small differences, such as the higher relative BoD of bladder and uterus-cervix cancers and the lower relative BoD of cancer of the pancreas in Spain.

Estimate of BoD due to cancer for the year 2000 has some limitations to describe the current situation. Advances in treatment and secondary prevention may have caused a reduction in mortality and an increase in duration of some cancers. However, given the lack of previous data of cancer BoD in Spain, data from 2000 are necessary to estimate changes over time and to assess the influence that medical advances may have had on mortality and disability. In the case of cancer, this would be feasible, given that mortality, incidence and survival data are being collected systematically.

One of the difficulties in calculating DALYs is the estimation of disability weights. Some studies have published weights [16,32] that can be used to estimate BoD. Alternatively, country-specific disability weights can be obtained. The choice of disability weights may affect the final results, in terms of absolute BoD and even the cancer ranking. One study [33] showed that disability weights did not have a conclusive influence on the number of DALYs lost due to breast cancer. However, disability weights influence could be greater in cancers with a high disability component. In addition, it could become greater if YLD component of BoD due to cancer were to increase as a consequence, say, of rises in survival. Taking these limitations into account, disability weights used in our study were based on those of the Dutch study [16], because they are differentiated by disease stage and calculated in a country with comparable characteristics to those of Spain. Indeed, they are regarded as a reference for developed countries and they are used in most BoD studies and this allows us to compare our results with theirs.

Another difficulty comes from the limited availability of reliable data sources for mortality, incidence and duration. Mortality data of Spain have good quality. In the case of incidence data, the main limitation is coverage of the tumour registries. In Spain there is not a National Registry of Cancer. Some regions have a population-based regional registry, which covers nearly all population in the region, but many other regions do not have it. As a result, the Spanish population covered by registries is approximately 25% [34]. Survival data classified by tumour stage would have allowed us to estimate disease duration more accurately, but they are not available. Overall, the quality of the data sources used can be regarded as adequate [14] and they are probably the most reliable data sources available at the time of this study. Consistency of incidence, mortality and duration data were checked and proved using DisMod II.

The BoD of some neoplasms may be underestimated [35] as a result of setting duration of disease at five years in the case of patients who were cured. Cancers that may be affected by this decision are mainly those that most frequently leave sequels, such as prostate, breast, stomach, oesophageal or colorectal cancers. The 5-year criterion was maintained because it is conservative and in line with the methodology of other studies [3,4,17,18,33].

Some other assumptions made in the disease model used in the calculation of DALYs, such as duration of some of the disease phases, are based on expert opinion. They are the same that are applied in other BoD studies [3,4]. This allows us to make comparisons between studies. Furthermore, given the lack of reliable data sources, we had to estimate cure rates from survival data to construct the disease model. Even though survival data from EUROCARE-3 study can be considered reliable, they provide some additional uncertainty to our BoD estimate. In order to help readers interpret our results, we have tried to describe all assumptions and calculations made. On top of that, we have provided bibliographic references containing this information.

Social values applied in BoD estimates, such as age-weighting and discount rate are another source of uncertainty and have been controversial issues. We have applied the values more frequently used in the literature. The age-weighting gives more relevance to deaths in young and middle age. Its main consequence is on the age distribution pattern of BoD. In our results the age weighting may have provided cancer sites more frequent in middle ages, such as breast, leukaemias, melanoma, brain and lymphomas with higher BoD. The 3% discount rate gives more weight to deaths that occur nearer to the present time. This assumption may increase the proportion of BoD due to YLD and decrease the BoD in children (although this influence would be more relevant in countries or diseases with high childhood mortality). Given that discount rate adds more weight to older ages and age weighting gives less weight to this age group, their joint influence on our results will not be crucial [36].

Some controversy exists about the relevance of making accurate estimates of the BoD for priority setting. It is generally accepted that many criteria should be taken into account in order to make an efficient allocation of resources. The main advantage of DALYs with respect to other epidemiological measures, such as mortality or prevalence, frequently considered when establishing priorities, is that DALYs provide a unified measure of mortality and disability. This could be useful to evaluate health interventions being focused on reducing mortality, incidence or both. In this sense, DALY estimates could be used as an outcome measure when conducting cost-effectiveness studies [37]. In addition, the proportion of YLD and YLL in different diseases can be used to suggest the most appropriate health interventions: in cases where YLL is predominant, those targeted at preventing the appearance of disease and in cases where YLD is predominant, those targeted at reducing sequelae and at enhancing quality of life.

## Conclusion

This study provides a global view of the epidemiological situation of cancer in Spain, reporting data on mortality, incidence, duration and disability of all leading malignant neoplasms. This may be useful to study the evolution of the burden of disease linked to different cancers and to analyse how this evolution affects mortality and disability components. It also highlights the need to increase the coverage of Spanish tumour registries and underscores the fact that, in addition to its associated mortality, cancer generates considerable disability.

## Competing interests

The authors declare that they have no competing interests.

## Authors' contributions

NFB participated in the design of the study and in the statistical analysis and drafted the manuscript. EAM conceived of the study, and participated in its design and coordination and helped to draft the manuscript. CMG participated in the design of the study and helped to draft the manuscript. RGM participated in the design of the study and helped to draft the manuscript. AG participated in the coordination of the study and helped to draft the manuscript. BPG participated in the design of the study and in the statistical analysis. GLA participated in the design of the study and in the statistical analysis. All authors read and approved the final manuscript.

## Pre-publication history

The pre-publication history for this paper can be accessed here:



## Supplementary Material

Additional file 1 **Classification of cancers at diagnosis and distribution of incident cases in subgroups.** Spain 2000. The table presents the percentage of patients in each subgroup at the time of diagnosis, for those cancer sites in which the disease model subdivided groups: breast, lung, melanoma, leukaemias, Hodgkin's disease and Non-Hodgkin's lymphoma. Click here for file

Additional file 2 **Cure thresholds and cure rates, by cancer site and sex.** Spain 2000. The table presents the cure thresholds assigned to each cancer site, and the cure rates that were used to calculate DALYs. These data were estimated from survival data from the EUROCARE-3 study. Click here for file
