# Supplementary material for: Burden of disease due to cancer in Spain
Source: BMC Public Health. 2009 Jan 30;9:42. doi: 10.1186/1471-2458-9-42 (PMC2642814; doi:10.1186/1471-2458-9-42)
Supplement: Additional file 1 — Classification of cancers at diagnosis and distribution of incident cases in subgroups. Spain 2000. The table presents the percentage of patients in each subgroup at the time of diagnosis, for those cancer sites in which the disease model subdivided groups: breast, lung, melanoma, leukaemias, Hodgkin's disease and Non-Hodgkin's lymphoma. [file 1471-2458-9-42-S1.doc]

**Additional file 1**

Classification of cancers at diagnosis and distribution of incident cases in subgroups. Spain 2000

| **CANCER SITE** | **CLASSIFICATION CRITERIA** | **SUBGROUPS** | **PERCENTAGES OF INCIDENT CASES** |
| --- | --- | --- | --- |
| Breast | Tumor size | Tumor size <2 cm | 16 |
|  |  | Tumor size 2-5 cm | 58 |
|  |  | Tumor size >5 cm | 26 |
| Lung | Histology and resectability | Small cell cancer | 23 |
|  |  | Operable, non-small cell cancer | 19 |
|  |  | Non operable, non-small cell cancer | 58 |
| Melanoma | Staging | No dissemination | 89 |
|  |  | Lymph node but no distant dissemination | 11 |
| Leukaemias | Histology | Acute Lymphoid Leukaemia | 11 |
|  |  | Acute Myeloid Leukaemia | 34 |
|  |  | Chronic Lymphoid Leukaemia | 38 |
|  |  | Chronic Myeloid Leukaemia | 17 |
| Hodgkin´s disease | Histology and resectability | Low grade, stages I and II | 3 |
|  |  | Low grade, stages III and IV | 30 |
|  |  | Intermediate/high grade, stage I | 14 |
|  |  | Intermediate/high grade, stages II, III and IV | 53 |
| Non-Hodgkin´s lymphoma | Histology and resectability | Low grade, stages I and II | 3 |
|  |  | Low grade, stages III and IV | 30 |
|  |  | Intermediate/high grade, stage I | 14 |
|  |  | Intermediate/high grade, stages II, III and IV | 53 |
