# Supplementary material for: Burden of disease due to cancer in Spain
Source: BMC Public Health. 2009 Jan 30;9:42. doi: 10.1186/1471-2458-9-42 (PMC2642814; doi:10.1186/1471-2458-9-42)
Supplement: Additional file 2 — Cure thresholds and cure rates, by cancer site and sex. Spain 2000. The table presents the cure thresholds assigned to each cancer site, and the cure rates that were used to calculate DALYs. These data were estimated from survival data from the EUROCARE-3 study. [file 1471-2458-9-42-S2.doc]

**Additional file 2**

Cure thresholds and cure rates, by cancer site and sex. Spain 2000

| **Cancer site** | **Cure threshold (years)** | **Cure rate (%)** | |
| --- | --- | --- | --- |
|  |  | **Males** | **Females** |
| Thyroid | 3 | 85 | 91 |
| Pancreas  Acute Lymphoid Leukaemia  Acute Myeloid Leukaemia  Kidney  Uterus and cervix  Brain | 5 | 5  34  22  58  -  20 | 6  42  16  61  74  16 |
| Stomach  Oesophagus  Colorectal  Liver  Gall bladder  Lung  Hodgkin´s disease  Non-Hodgkin´s lymphoma | 6 | 26  12  50  7  18  12  73  52 | 26  12  54  7  18  15  71  49 |
| Ovary  Bladder | 7 | -  68 | 39  65 |
| Chronic Lymphoid Leukaemia  Chronic Myeloid Leukaemia | 8 | 41  26 | 43  26 |
| Melanoma | 9 | 64 | 87 |
| Breast  Prostate | 10 | -  37 | 61  - |
| Myeloma | 12 | 8 | 8 |
